# Native trees show conservative water use relative to invasive trees: results from a removal experiment in a Hawaiian wet forest

**DOI:** 10.1093/conphys/cou016

**Published:** 2014-05-17

**Authors:** Molly A. Cavaleri, Rebecca Ostertag, Susan Cordell, Lawren Sack

**Affiliations:** 1 School of Forest Resources and Environmental Science, Michigan Technological University, 1400 Townsend Drive, Houghton, MI 49931, USA; 2 Biology Department, University of Hawaii at Hilo, HI 96720, USA; 3 Institute of Pacific Islands Forestry, USDA Forest Service, Hilo, HI 96720, USA; 4 Department of Ecology and Evolutionary Biology, University of California, Los Angeles, CA 90095, USA

**Keywords:** Invaded forest, invasive species removal experiment, lowland wet forest, *Metrosideros polymorpha*, sap flux, transpiration

## Abstract

While the supply of freshwater is expected to decline in many regions in the coming decades, invasive plant species, often ‘high water spenders’, are greatly expanding their ranges worldwide. In this study, we quantified the ecohydrological differences between native and invasive trees and also the effects of woody invasive removal on plot-level water use in a heavily invaded mono-dominant lowland wet tropical forest on the Island of Hawaii. We measured transpiration rates of co-occurring native and invasive tree species with and without woody invasive removal treatments. Twenty native *Metrosideros polymorpha* and 10 trees each of three invasive species, *Cecropia obtusifolia*, *Macaranga mappa* and *Melastoma septemnervium*, were instrumented with heat-dissipation sap-flux probes in four 100 m^2^ plots (two invaded, two removal) for 10 months. In the invaded plots, where both natives and invasives were present, *Metrosideros* had the lowest sap-flow rates per unit sapwood, but the highest sap-flow rates per whole tree, owing to its larger mean diameter than the invasive trees. Stand-level water use within the removal plots was half that of the invaded plots, even though the removal of invasives caused a small but significant increase in compensatory water use by the remaining native trees. By investigating the effects of invasive species on ecohydrology and comparing native vs. invasive physiological traits, we not only gain understanding about the functioning of invasive species, but we also highlight potential water-conservation strategies for heavily invaded mono-dominant tropical forests worldwide. Native-dominated forests free of invasive species can be conservative in overall water use, providing a strong rationale for the control of invasive species and preservation of native-dominated stands.

## Abstract (precis)

To gain understanding about invasive species physiology and also potential water conservation strategies, we investigated tree-level water use following a woody invasive removal experiment in Hawaii. Invasives had much higher water use rates than the native tree species, and stand level water use decreased by ∼50% after invasive removal.

## Introduction

Water conservation has long been of global concern, and invasive plant species are encroaching into and altering the ecohydrology of much of the Earth's terrestrial surface as a direct result of an increasingly mobile human population (Millennium Ecosystem Assessment, 2005). By definition, a non-native (alien) species is invasive if it successfully reproduces, forms self-replacing populations far from parent plants and has the potential to spread across large areas (Richardson *et al.*, 2000; Richardson and Pyšek, 2006; Rejmanek *et al.*, 2013). Species invasiveness has also been attributed to organismal competitiveness and, in undisturbed systems, successful competitors will often use limiting resources more efficiently or more quickly than co-occurring natives (Rejmanek *et al.*, 2013). As such, non-native invasive plants are often assumed to use more water than co-occurring natives, given evidence that invasives generally have faster growth rates (Cronk and Fuller, 1995; Pyšek and Richardson, 2007; van Kleunen *et al.*, 2010), higher rates of photosynthesis and higher rates of leaf-level water use (Leishman *et al.*, 2007; Cavaleri and Sack, 2010). Some studies have found that introduced tree species are more efficient at using water than native trees (e.g. Wise *et al.*, 2011); however, it is often not appropriate to extrapolate trends in leaf-level water use to whole-plant transpiration rates. For example, a global meta-analysis found that invasives showed significantly greater levels of water use at the leaf level, but that co-occurring invasive and native species were equally likely to have higher transpiration rates at the whole-plant scale (Cavaleri and Sack, 2010). This disconnect between results found at the leaf level vs. the whole-plant level may be due to artifacts of leaf-chamber measurements, variations in canopy structure, sapwood:leaf area ratios, plant size or age (Cavaleri and Sack, 2010). In addition to potential inconsistencies between measurements at various spatial scales, the vast majority of these studies have been conducted at the species level rather than the ecosystem (e.g. forest stand) or landscape level (Cavaleri and Sack, 2010). Taken together, these deficiencies reveal a need for more information about the water use of invasive and native plant species from the ecosystem perspective.

Invasive species removal experiments can help us to understand not only the ecological effects of invasive species, but also the restoration potential of an ecosystem (Zavaleta *et al.*, 2001; Díaz *et al.*, 2003). Removal experiments have been shown to increase overall diversity (e.g. Biggerstaff and Beck, 2007), but may also result in re-establishment of removed species or establishment of a new set of non-native species (Ehrenfeld, 2003; Adams and Galatowitsch, 2008; Elgersma *et al.*, 2011). In addition to answering questions about diversity, removal experiments can also provide critical baseline information about the water use of native species and the potential effects on overall system water balance after invasive species removal.

Woody invasives can greatly impact the water balance of a system, as woody plants generally have higher rainfall interception, higher transpiration rates, deeper roots, greater standing biomass, greater carbon sequestration and longer growing seasons than co-occurring herbaceous plants (McNaughton and Jarvis, 1983; Enright, 2000; Calder and Dye, 2001; Farley *et al.*, 2005; Huxman *et al.*, 2005). Investigations of hydrology after removal of invasive woody species have shown increased water yield (Dye and Poultera, 1995; Prinsloo and Scott, 1999; Dye and Jarmain, 2004), reduction in evapotranspiration (Cleverly *et al.*, 2006), rise in water table (Asbjornsen *et al.*, 2007) and reduction in amplitude of groundwater fluctuation (Martinet *et al.*, 2009). However, many experiments have also shown no evidence of a change in water yield (Doody *et al.*, 2011; Moore and Owens, 2012), a return to baseline rates in the long term as a result of recolonization (Hatler and Hart, 2009; Doody and Benyon, 2011) or even increased stand evapotranspiration after removal due to compensatory water use by remaining native species (Moore and Owens, 2012). Whether or not invasive species removal results in increased water yield over time depends greatly on the particular species involved and site-specific conditions, such as whether the invading species are displacing native species or invading a previously unoccupied niche (Dye and Jarmain, 2004; Doody and Benyon, 2011; Doody *et al.*, 2011). One way to promote water conservation is the preservation and restoration of forests dominated by native species that exhibit conservative water use.

Historically, non-native trees were planted extensively throughout the Hawaiian Islands for timber and/or to mitigate the negative effects of erosion (Cox, 1999). Many of these non-native species, especially the fast-growing ‘pioneers’, have become invasive and have subsequently displaced native species throughout the islands (Vitousek, 1986; Denslow, 2003). The effects of these invasive trees on the hydrology of Hawaiian ecosystems are largely unknown. Hawaiian soils are generally formed of young lava parent materials and, as a result, are quite thin, with very low water-holding capacities (Natural Resources Conservation Service, NRCS, 2012). Therefore, water may be limiting to tree growth or seedling survival even in lowland wet forests, and invasives may compete with natives for this resource under certain environmental conditions (Schulten *et al.*, 2014). While mean annual precipitation in Hawaiian tropical rain forests is high, recent analyses of rainfall records imply that periods of short-term drought are common (J. D. Michaud, unpublished). Additionally, predictions suggest that rainfall and streamflow of the Hawaiian islands will decrease with climate change (Oki, 2004; Timm and Diaz, 2009), which will also have great implications for reduced aquifer recharge. Native ‘ōhi’a (*Metrosideros polymorpha*)-dominated and *M. polymorpha*–*Acacia koa* mixed forests represent 38% of Hawaii Island's total forest cover (Asner *et al.*, 2011); therefore, species conversion away from this important species has the potential greatly to affect aquifer recharge across the islands if the invaders use water at higher rates.

In order to determine the potential effects of large-scale invasive species removal on the hydrology and ecology of a heavily invaded *M. polymorpha*-dominated lowland wet forest in Hawaii, all invasive species were removed from four 100 m^2^ plots, which were paired with four invaded plots, where both native and invasive tree species were left intact. Environmental changes and restoration potential were examined in the years following removal. Earlier findings show that native seedling establishment was severely hindered by invasives and that removal treatments greatly improved native seedling establishment (Cordell *et al.*, 2009). Higher soil water availability was measured in removal plots, but no change in growth rates of native species occurred after invasives were removed (Ostertag *et al.*, 2009). These previous results indicated that native species were more conservative in resource use in general, and thus may not respond strongly in growth to invasive canopy removal, at least at the adult stage. However, whether natives were in fact using more water once invasives had been removed remained to be tested.

We used a heavily invaded monodominant *M. polymorpha* forest as a model for conservative native-dominated tropical forests being invaded with potentially faster-growing, higher water-using invasive woody species. Our objective for this study was to investigate the effects of removal of invasive tree species on both remaining native tree transpiration rates and stand-level transpiration in a heavily invaded Hawaiian lowland wet forest. Our specific hypotheses were threefold: (i) *M. polymorpha* is more conservative in water use than three co-occurring invasive tree species (*Cecropia obtusifolia*, *Macaranga mappa* and *Melastoma septemnervium*); (ii) removal of invasive species will not dramatically increase transpiration rates of remaining *Metrosideros*; and (iii) at stand level, removal of invasives will result in a significant decrease in total plot transpiration, thus an increase in potential aquifer recharge. With this unique study design, we aim to gain insight into comparative water use of co-occurring native and invasive tree species, to investigate the effects of removal of invasive woody species on the hydrology of the stand and to provide information to managers about potential water-conservation strategies for heavily invaded mono-dominant wet tropical forests.

## Materials and methods

### Site description

This study was conducted in a 43.3 ha lowland wet forest (Price *et al.*, 2007) on the eastern coast of Hawaii Island at an elevation of 30 m. The forest, located near the Hilo Airport within the Keaukaha Military Reservation (19°42.15 N, 155°2.40 W), was fenced in 2002 to exclude feral pigs. The site receives ∼3300 mm rainfall per year, and annual potential evapotranspiration is 3026 mm using the Penman–Monteith method (Giambelluca *et al.*, 2014). Rainfall is relatively aseasonal; long-term weather data from the Western Regional Climate Center show slightly higher rainfall rates in the months of March and November (∼350 mm month^−1^), but no month receives <100 mm of rain total (www.wrcc.dri.edu/summary/climsmhi.html). The lowland wet forest is located on a 750- to 1500-year-old ‘a’ā lava flow and has a mean annual temperature of 23.2°C (National Weather Service, Hilo International Airport). Soils are isohyperthermic Typic Udifolists of the Papai Series, which describe well-drained, cobbly soils overlying the lava (Ostertag *et al.*, 2009). The forest overstory (23–35 m) is dominated by native ‘ōhi’a (*M. polymorpha*) and lama (*Diospyros sandwicensis*), a forest type found only on the eastern side of Hawaii Island (Gagné and Cuddihy, 1999). While the overstory is largely native, this forest has been heavily invaded, and the younger stems are primarily non-native. The most notable invasive tree species of this forest include trumpet tree (*C. obtusifolia*), bingabing (*M. mappa*), strawberry guava (*Psidium cattleianum*) and Indian rhododendron (*M. septemnervium*; Zimmerman *et al.*, 2008). The Hawaii Weed Risk Assessment scores for these species range from 8 to 18, where scores >6 indicate high risk of invasiveness (hpwra.org). Native woody species with DBH (stem diameter at breast height, or 1.3 m) of >2 cm have a basal area of 19.8 m^2^ ha^−1^ and stem density 1591 ha^−1^, while non-native species have similar basal area (16.4 m^2^ ha^−1^) but an order of magnitude higher stem density (17 199 ha^−1^; Zimmerman *et al.*, 2008). Without active intervention, non-native tree species will soon dominate the overstory as well as the understory of this rare native forest type.

### Experimental design

The full experimental design included four pairs of 10 m × 10 m invaded forest plots and invasive removal plots for investigation of germination, seed ecology, productivity, microclimate, decomposition, leaf traits and soil nutrients (Cordell *et al.*, 2009; Ostertag *et al.*, 2009). We measured sap flux in two invaded plots and two removal plots. It was not logistically feasible to perform sap-flux measurements pre- and post-treatment due to the large disturbance caused by harvesting over half of the basal area in the removal plots. Paired invaded and removal plots were located ∼20 m apart. In April–June 2004, all non-native tree species were removed mechanically from the removal plots, leaving only native species. Small plants were hand-pulled, trees were harvested with saws, and cut stumps were dosed with herbicide (Garlon 4; Dow AgroSciences LLC). After harvest, removal plots contained only mature trees and seedlings, as there were few native mid-story species present (with the occasional exception of *Pandanus tectorius* and *Psychotria hawaiiensis*). All non-native species were also removed from a 2.5-m-wide buffer around each 10 m^2^ removal plot. Detailed information on the biomass of each species removed can be found in the article by Ostertag *et al.* (2009).

Four species across the two pairs of plots were instrumented for sap-flux measurements: one native species, *M. polymorpha*; and three non-native invasive species, *C. obtusifolia*, *M. mappa* and *M. septemnervium*. Hereafter, species will be referred to by their genus names. Together, these four species represented ∼94% of basal area and 89% of the stems for the stand. Five individuals of *Metrosideros* were instrumented in each of the four plots (20 native trees in total), and five individuals each of invasive *Cecropia*, *Macaranga* and *Melastoma* were instrumented in each of the two invaded plots (10 trees per invasive species), for a total of 50 trees instrumented overall.

### Measuring tree sapwood area

Prior to sap-flux sensor installation, DBH-to-sapwood thickness allometric relationships were developed by measuring trees within the same stand, but outside the experimental plots. In October 2007, 30 trees each of *Macaranga* and *Cecropia* and 20 trees each of *Metrosideros* and *Melastoma* were cored using a 4.3-mm-increment borer (Haglöf, Sweden), and DBH was measured. Trees were selected along nearby transects outside the experimental plots. Every 5 m along each transect, the nearest tree of the specified species was selected for coring. We avoided coring trees within the experimental plots because they were also part of a larger study, in which tree growth was measured continuously. Within 24 h of collection, cores were stained with triphenyl tetrazolium chloride in a phosphate buffer solution (Sigma-Aldrich, St Louis, MO, USA) to identify the living parenchymal cells of the sapwood layer (Spicer and Holbrook, 2005). After soaking for 24 h in triphenyl tetrazolium chloride solution, each core was inspected visually (unmagnified) and measured for thickness of bark, phloem, sapwood and heartwood. For each tree instrumented for sap flux, sapwood and heartwood thickness were estimated using species-specific allometric relationships between DBH and sapwood thickness (Table 1). The total cross-sectional area of the sapwood annulus (*A*_sw_; in square centimetres) was calculated for each instrumented tree using the following formula:

(1) $${A_{{\rm sw}}}={\pi}{({r_{{\rm sw+hw}}})^2} - {\pi}{({r_{{\rm hw}}})^2}$$

where *r*_sw+hw_ is the radial thickness of the sapwood plus the heartwood, and *r*_hw_ is the radial thickness of the heartwood (all in centimetres).

**Table 1:** Allometric relationships between stem diameter at breast height (DBH) and sapwood thickness for each species instrumented

Species	β_0_	β_1_	DBH (range; cm)	*R*^2^	*n*
*Metrosideros polymorpha*	2.257	0.018	7.1–45.1	0.25	20
*Cecropia obtusifolia*	−0.430	0.190	7.5–34.4	0.60	30
*Macaranga mappa*	−1.300	0.460	6.2–27.2	0.84	30
*Melastoma septemnervium*	0.734	0.236	5.1–7.6	0.20	20

Coefficients are provided for the following linear relationship: sapwood thickness (in centimetres) = β_0_ + β_0_ × DBH (in centimetres), along with *R*^2^ values and number of trees sampled to develop each species-specific allometry (*n*).

### Measurement of sap flux

We measured sap flux for 10 months, from February to November 2008, using modified Granier-style variable-length heat-dissipation probes (James *et al.*, 2002). Each sensor consisted of two probes, an unheated control probe and a continuously heated downstream (upper) probe. Probes were tipped with 1-cm-long aluminum tubes, each containing copper–constantan differential thermocouples (PFA-teflon coated wires, 0.12 mm diameter; Omega Engineering, Stanford, CT, USA), while the heated probe also contained a coiled length of nichrome wire with a final resistance of ∼100 Ω (Rediohm-675, 436 Ω m^−1^; H.P. Reid, Palm Coast, FL, USA). For each sensor, the differential voltage between the two probes was measured every minute, and 10 min averages were logged with AM 16/32 multiplexers connected to CR1000 dataloggers (Campbell Scientific, Logan, UT, USA) powered by 7 Ah 12 V sealed lead–acid batteries (Radio Shack, Fort Worth, TX, USA). Sap-flux density (ν; in grams of water per metre squared sapwood area per second, g m^−2^_sw_ s^−1^) was calculated for each sensor for each 10 min mean voltage using the calibration originally developed by Granier (1985) and specifically calibrated for this probe design (McCulloh *et al.*, 2007):

(2) $$\nu=119 \times {\left( {\displaystyle{{\Delta {V_{\rm m}} - \Delta V} \over {\Delta V}}} \right)^{1.231}}$$

where Δ*V* is the difference in voltage between the two thermocouples for each 10 min interval, and Δ*V*_m_ is the daily maximal difference in voltage, corresponding to minimal daily sap flow. The difference in voltage between the two probes is proportional to the difference in temperature, which decreases as sap flux increases in velocity due to thermal dissipation of the heated downstream probe.

The heated probes were powered by a constant current through an adjustable voltage regulator, set to supply 0.15 W per sensor. Continuous power was supplied to all probes by six 12 V deep cycle marine batteries (ACDelco, Grand Blanc, MI, USA), which were recharged with four 115 W solar panels (Evergreen Solar, Marlborough, MA, USA) connected to a 20 A charge controller (Morningstar SunSaver SS-20L-12V, Washington Crossing, PA, USA). At this site, only a small gap in tree cover was available for solar panel installation (created by cutting down several trees next to the road), and it was extraordinarily wet and cloudy for much of the time. As a result, solar power was not sufficient to recharge the batteries unless the probes were turned off every second day, allowing the batteries to recharge on off-days. While this enabled battery power to keep up with probe power draw, it also created spurious data after probes were turned back on until trees and probes equilibrated. Thus, only data from 04.00 to 20.00 h were used in all further analyses.

To account for variability in sap flux with sapwood depth, a subset of three trees per species were instrumented with multiple sensors installed at each centimetres of estimated sapwood depth (two to five probes per tree, depending on tree DBH). Sapwood depth was estimated for all instrumented trees using the species-specific DBH allometric equations described above (Table 1). The remaining trees were instrumented with one sap-flux sensor each in the outermost 1 cm annulus of sapwood. Sensors were positioned at or near 1.3 m in height, and in the subset of trees instrumented at variable depths; multiple sensors were offset vertically and horizontally from each other to avoid heating control probes. All sensors were wrapped with reflective insulation to protect from external thermal gradients (Reflectix, Markleville, IN, USA). Patterns in peak sap-flux values across sapwood depth were calculated in absolute terms and also relativized as a proportion of the sap-flow rate of the outermost sapwood annulus for the subset of trees measured at multiple sapwood depths. The relativized values were used to gap fill by species for the rest of the trees that were instrumented only for sap-flow measurement in the outermost layers of sapwood.

### Sap-flux parameters and statistical analyses

Sap-flux density per unit sapwood area (in grams of water per metre squared sapwood area per second) was calculated using Equation 2 for each 10 min interval for each sensor. To assess differences in sap-flux rates with sapwood depth (i.e. radial variation) within species, mean diurnal traces of sap-flux density were calculated for each depth by averaging 10 min values of sap-flux density across all 10 months of data. Peak daily sap-flux density (in grams of water per metre squared sapwood area per second) was calculated as the maximal daily value of *v* for each probe, and these values were used to compare radial variation in peak sap-flux density. To account for this radial variation in whole-tree values of sap flow, we divided each tree into concentric circles of sapwood annuli (one per centimetre of sapwood thickness) using DBH–sapwood allometries described above, and then determined sap-flux density for each annulus. For trees with only one sensor, we estimated sap-flux density for each deeper annulus as a fraction of the measured sap-flux density of the outermost annulus. Fractions used in the gap-filling procedure were based on mean peak daily sap-flux data from the subset of trees that were instrumented at multiple depths. Each 1-cm-thick annulus (both measured and estimated) was defined by an outer radius (*r*_*i*_) and an inner radius (*r*_*i*−1_). We calculated sap flow for each annulus by multiplying sap-flux density at a specified depth (*v*_*i*_) by the area of the corresponding annulus. For each tree, these annulus-based sap-flux rates were summed for *n* annuli to yield whole-tree sap-flow rates for each 10 min interval (*Q*; in grams per tree per second):

(3) $$Q=\mathop \sum \limits_{i=1}^n {\pi}(r_i^2 - r_{i - 1}^2 ){v_i}$$

Whole-tree sap flow (*Q*) was then divided by whole-tree sapwood area (estimated using DBH–sapwood allometry) to yield sap-flow rates per unit sapwood area for each tree for each 10 min interval (in grams of water per metre squared sapwood area per second). Means and standard errors of these 10 min values across all 10 months of data were calculated to plot diurnal curves by species and treatment. These sapwood area-specific rates were also used to calculate peak daily sap-flow rates (*E*_max_, in grams of water per metre squared sapwood area per second) for species and treatment comparison. Total daily water use per unit sapwood area (*E*_tot/SW_, in kilograms of water per metre squared sapwood area per day) and per unit tree (*E*_tot/Tr_, in kilograms of water per tree per day) were calculated by summing under the diurnal curves.

To estimate plot-level water use by species, total daily water use per unit tree (*E*_tot/Tr_) by DBH was plotted, and exponential growth models were fitted for each species (*E*_tot/Tr_ = exp(*a* × DBH), where *a* is a fitted species-specific constant. Plot-level water use was calculated by estimating water use of all trees >1 cm DBH in each plot (including trees not instrumented for sap flow) using the DBH of every tree and estimating *E*_tot_ for each tree by fitting species-specific exponential models predicting *E*_tot/Tr_. For species in the plots that were represented by our sap-flux data, an exponential model was fitted across all data for non-native species pooled to simulate an overall average non-native species relationship between *E*_tot_ and DBH. The same procedure was performed for native species that were not instrumented for sap flux.

Daily parameters *E*_max_, *E*_tot/Tr_ and *E*_tot/SW_ were compared across species and treatment using one-way analysis of variance procedures (Sokal and Rohlf, 1995) to determine species differences of co-occurring native and invasive species within the invaded plots and the effects of non-native removal on the native species. Tukey's HSD comparisons were used for *post hoc* mean separation. All statistical analyses were performed with SAS version 9.1 (SAS Institute, Inc., Cary, NC, USA), with α = 0.05.

## Results

### Contrasts between stand characteristics of invaded forest plots and invasive removal plots

**Figure 1:**
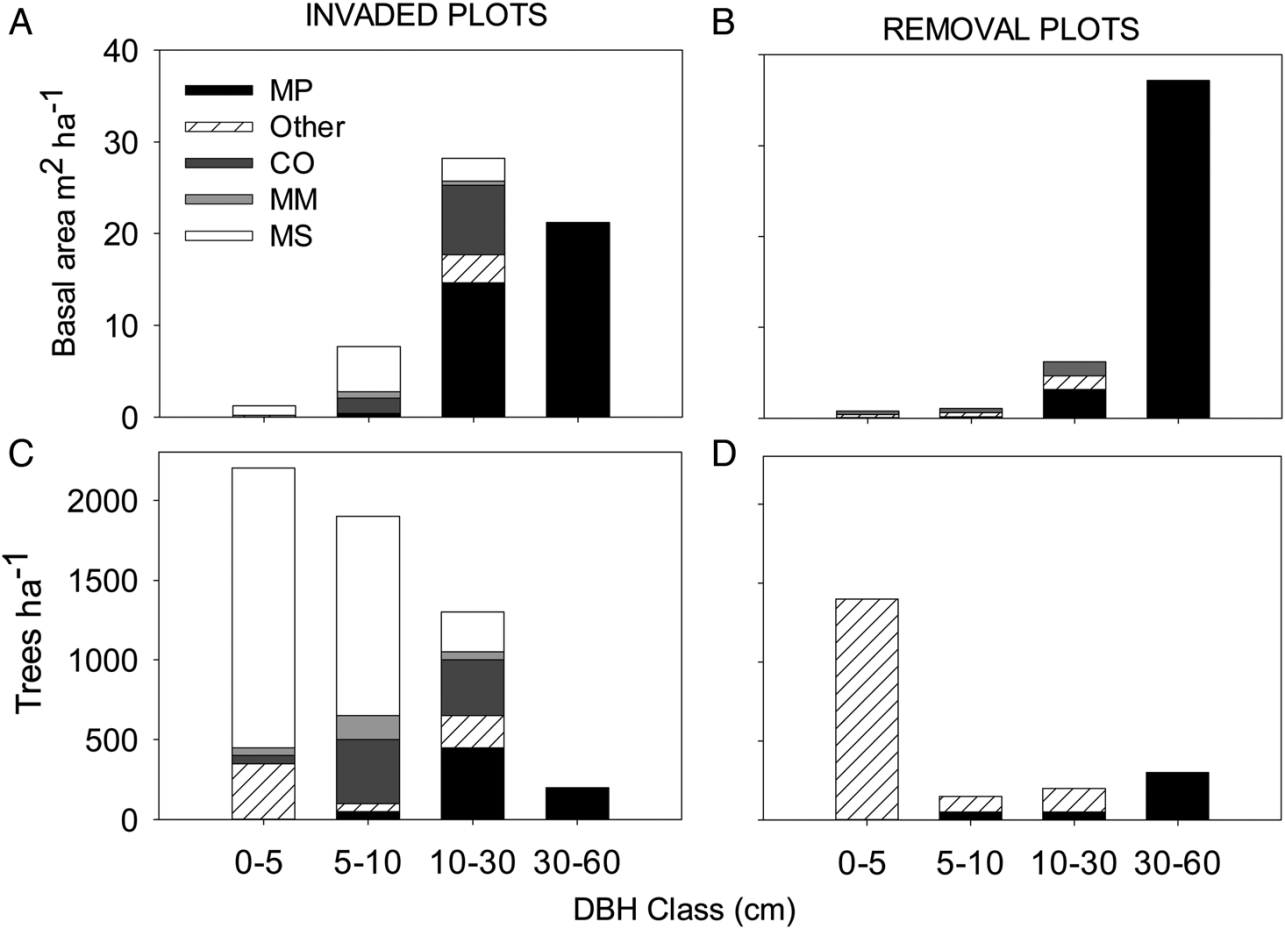
Basal area and trees per hectare by species and tree diameter at breast height (DBH) class, summed over invaded plots (**A** and **C**) and removal plots (**B** and **D**). Here and for subsequent plots, species codes are as follows: native *Metrosideros polymorpha* (MP), and invasives *Melastoma septemnervium* (MS), *Macaranga mappa* (MM) and *Cecropia obtusifolia* (CO). ‘Other’ represents both native and invasive species not quantified in this study.

Vegetation removal increased light transmittance (Wong, 2007) and soil temperatures, while it reduced humidity, leaf area indices, litterfall and nutrient inputs (Ostertag *et al.*, 2009). Instrumented *Metrosideros* ranged in DBH from 9 to 42 cm, while DBH ranges for invasive *Cecropia*, *Macaranga* and *Melastoma* were 7–27, 6–17 and 7–19 cm, respectively. Invaded forest plots contained lower basal area in invasives (45.3% of total; Fig. 1A) than the native species, but invasives had much higher stem density (91.5% of total) than *Metrosideros* (Fig. 1C). The stem density of natives was similar in both removal and invaded plots, but the basal area was slightly higher in the removal plots owing to larger trees (Fig. 1). Invasive *Melastoma* had the highest stem density of all species, but still maintained low basal area due to many small trees (Fig. 1A and C). The large difference in stem density of small-diameter ‘other’ species in the removal plots was primarily accounted for by small kopiko saplings (*P. hawaiiensis*), a native species which was not as well represented in the invaded plots.

### Variability in sap flow with sapwood depth

**Figure 2:**
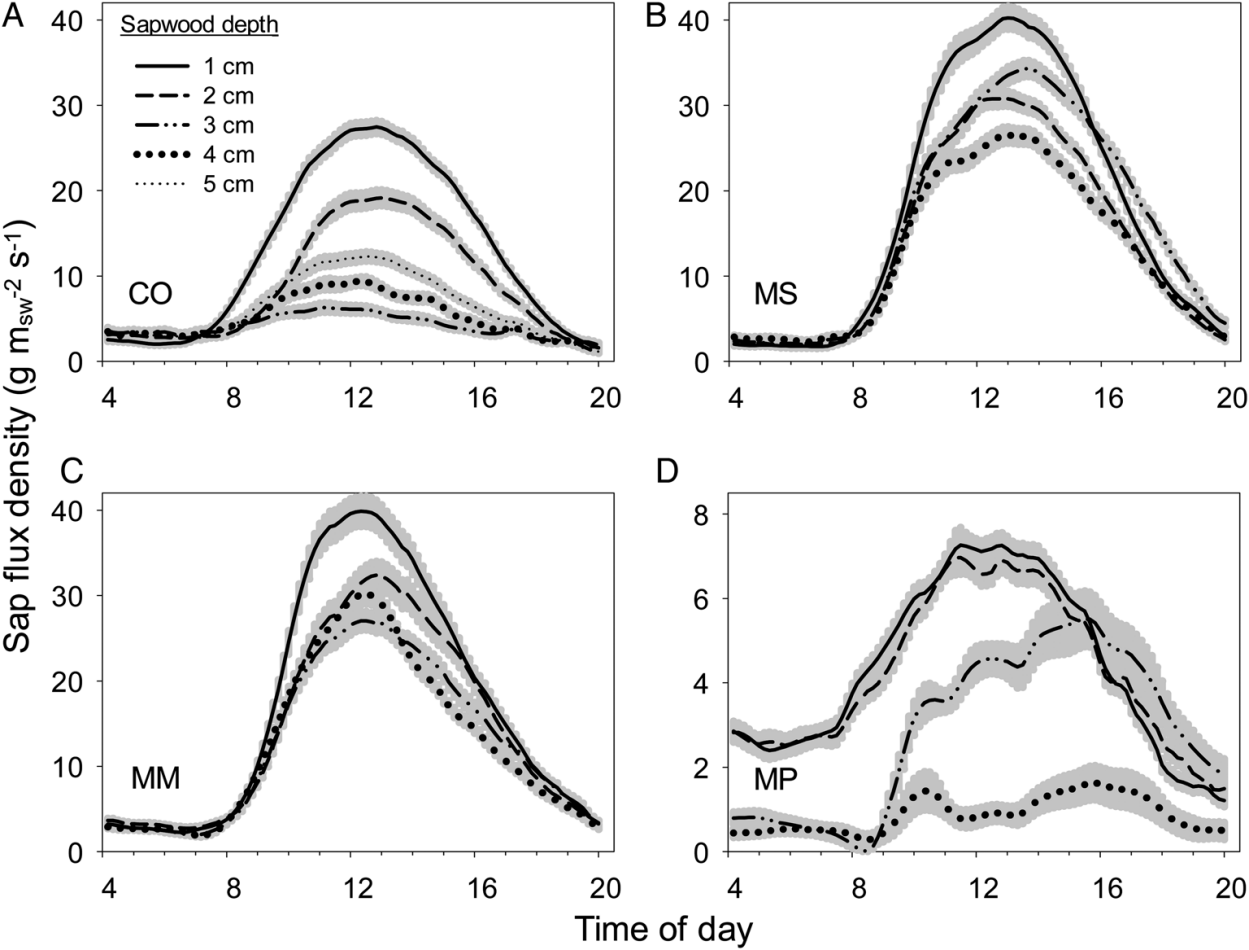
Mean 10 min values of sap-flux density per unit sapwood area by sapwood depth (probe depth) for each species (**A**–**D**), averaged over 10 months. Data represent a subset of 2–3 trees per species where sap-flux probes were installed at multiple depths. Grey shading around lines represents standard error of the mean 10 min value. Note the change in scale for *Metrosideros* (MP; plot D). See legend to Fig. 1 for species codes.

**Figure 3:**
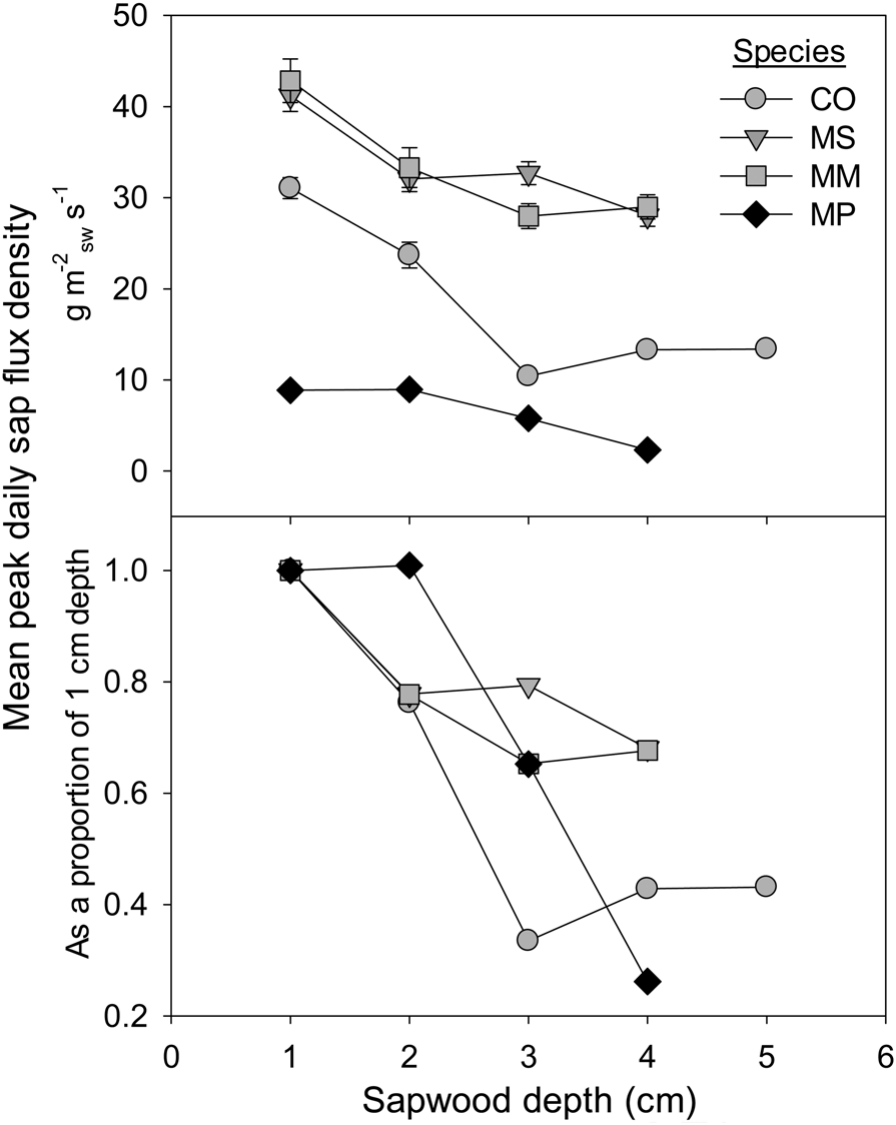
Mean peak daily sap-flux density per unit sapwood area (top panel) by sapwood depth for each species. The bottom panel represents the same data, but each value is displayed as a proportion of the sap-flux rates in the outermost sapwood depth (1 cm). Data represent a subset of two to three trees per species where sensors were installed at multiple depths. Data from the bottom panel are used to gap fill sap-flux rates in trees with only one sap-flux probe in the outer annulus. See legend to Fig. 1 for species codes.

Sap-flow rates per unit sapwood area decreased with sapwood depth for all species, with all three invasive species, *Cecropia*, *Melastoma* and *Macaranga*, showing the steepest declines between the outermost 1 and 2 cm depths (Figs 2 and 3). *Metrosideros* showed much smaller variation in sap flux with depth overall, and total rates for all depths were small relative to the three invasive species (note the different *y*-axis scale in Fig. 2D). Additionally, while sap flux was not measured throughout the entire night owing to methodological artifacts, there appears to be some evidence of low-level nocturnal water uptake by all species, because early morning hours of sap-flux density (e.g. between 04.00 and 07.00 h) were non-zero (Fig. 2).

### Differences in sap flow between *Metrosideros* and invasive species across invaded and removal plots

**Figure 4:**
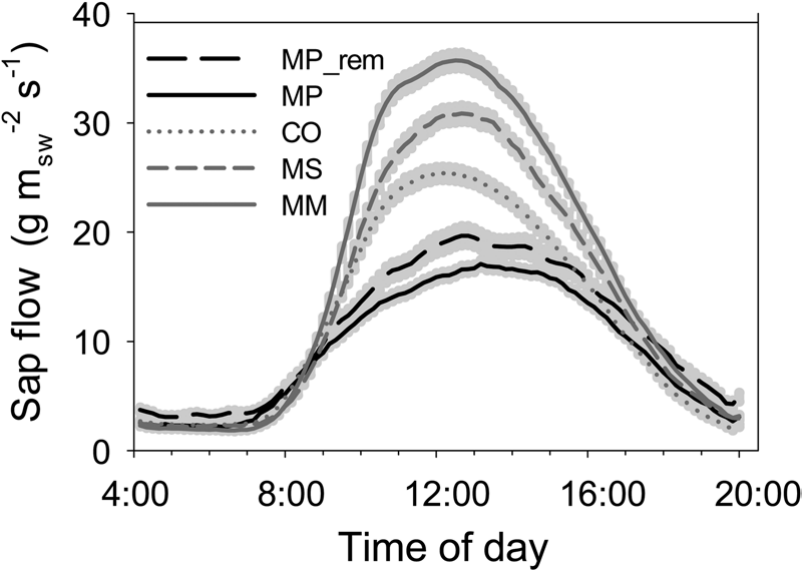
Mean 10 min values of sap flow per unit sapwood area by species and treatment, averaged over 10 months. Data represent all instrumented trees. MP_rem represents *Metrosideros* from the removal plots. Grey shading around lines represents standard error of the mean 10 min values. See legend to Fig. 1 for species codes.

**Figure 5:**
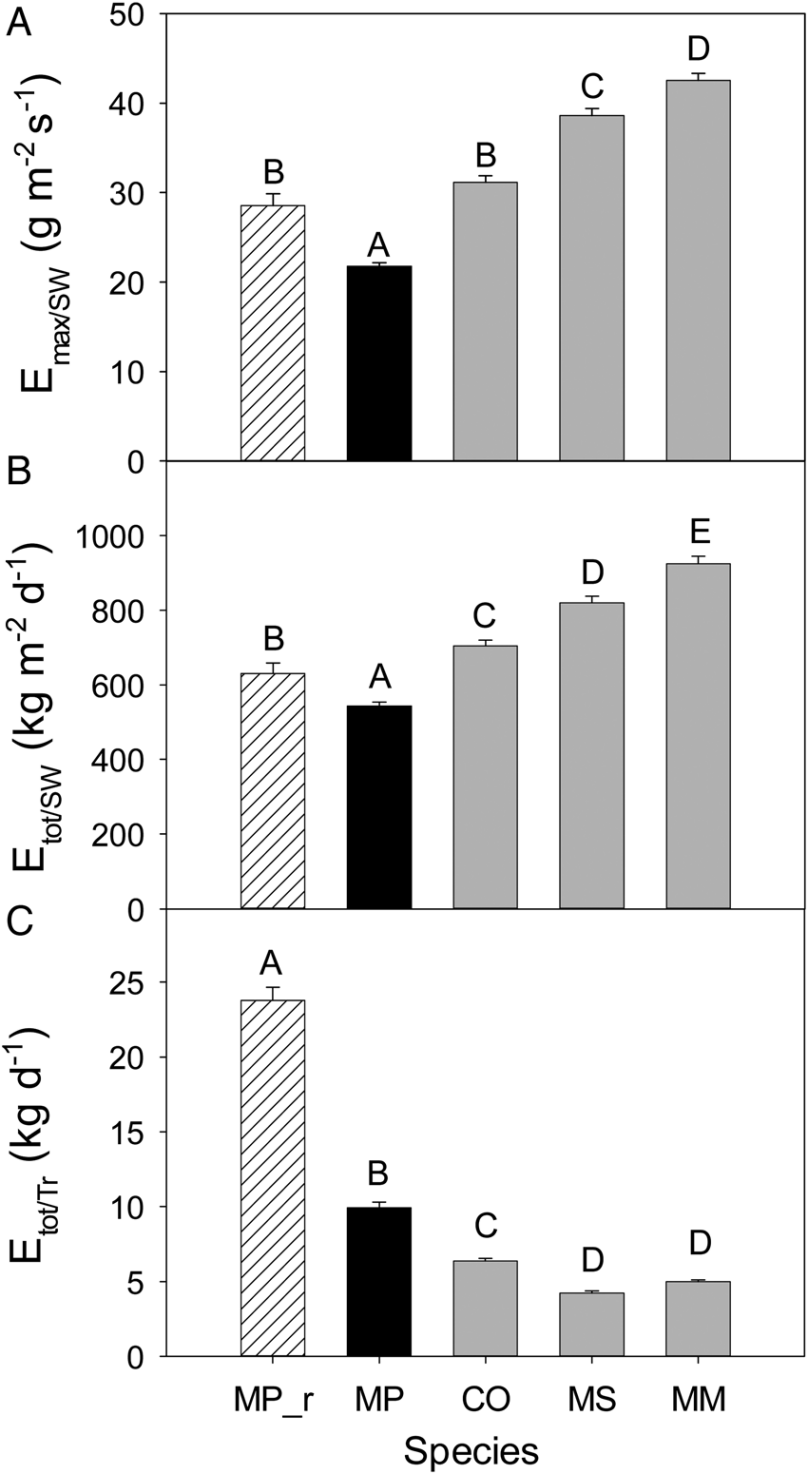
Mean peak daily sap flow per unit sapwood area (**A**), mean total daily sap flow per unit sapwood area (**B**) and mean total daily sap flow per tree by species and treatment (**C**). MP_rem represents *Metrosideros* from the removal plots. Error bars represent standard errors, and letters indicate statistical differences between means based on Tukey's HSD comparisons (α = 0.05). See legend to Fig. 1 for additional species codes.

**Figure 6:**
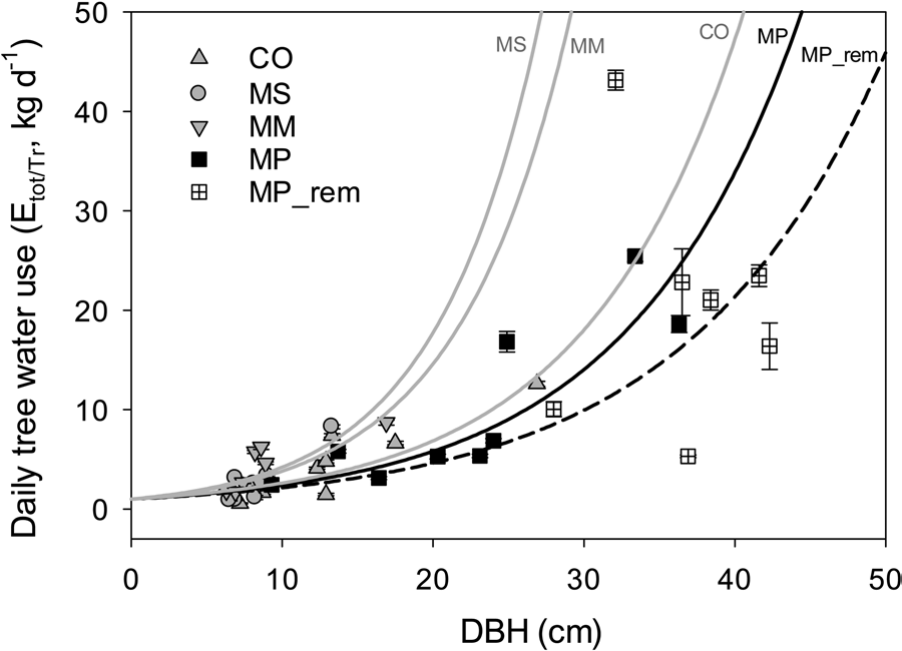
Mean daily water use per tree for each tree instrumented, averaged over 10 months by tree diameter at breast height (DBH). Data represent all trees. MP_rem represents *Metrosideros* from the removal plots. See legend to Fig. 1 for additional species codes. For each species, data were fitted with exponential growth models [water use = exp(*a* × DBH)]. Exponential formulas were used to estimate daily tree water use for trees not instrumented with sap-flow probes in order to estimate total water use per species per plot. Species-specific *a* coefficients and *R*^2^ values for each fitted line are as follows: *Cecropia* (CO), *a* = 0.0964, *R*^2^ = 0.78; *Melastoma* (MS), *a* = 0.1438, *R*^2^ = 0.67; *Macaranga* (MM), *a* = 0.1341, *R*^2^ = 0.52; *Metrosideros* (MP), *a* = 0.0880, *R*^2^ = 0.7131; and *Metrosideros* from removal plots (MP_rem), *a* = 0.0765, *R*^2^ = 0.42.

Diurnal curves of each species (each 10 min interval value was averaged over the 10 month data set) showed differences in magnitude among species and also slight differences in the timing of peak flow between species. Invasive *Cecropia* reached peak sap flow earliest, around midday, whereas sap-flow rates of the other species tended to peak later in the afternoon, around 13.00–14.00 h (Fig. 4). Within the invaded plots, invasive species all had greater peak daily sap flow and total daily water use per unit sapwood area, with *Macaranga* showing the highest rates, followed by *Melastoma* then *Cecropia* (Fig. 5A and B). *Metrosideros* in the removal plots also had higher peak sap-flow rates and total daily water use per unit sapwood area than *Metrosideros* in the invaded plots (Fig. 5A and B). Total daily water use per tree, however, was substantially higher for *Metrosideros* in the removal plots and slightly higher in the invaded plots than for the invasive species, because the *Metrosideros* trees were much larger and therefore transpired more water per tree than the average invasive tree (Fig. 5C). When tree size was taken into account, and total daily water use per tree was plotted by DBH, all three invasives increased daily water use more steeply as trees grew larger (Fig. 6). The large difference in *E*_tot_ per tree for the natives in the removal plot vs. the natives in the invaded plot (Fig. 5C) was due to one influential outlier tree with very high sap-flow rates compared with the rest of the *Metrosideros* trees (Fig. 6). The exponential relationships between daily tree water use and DBH by species and plot census data were used to extrapolate tree water use to the plot level.

### Plot-level water use

**Figure 7:**
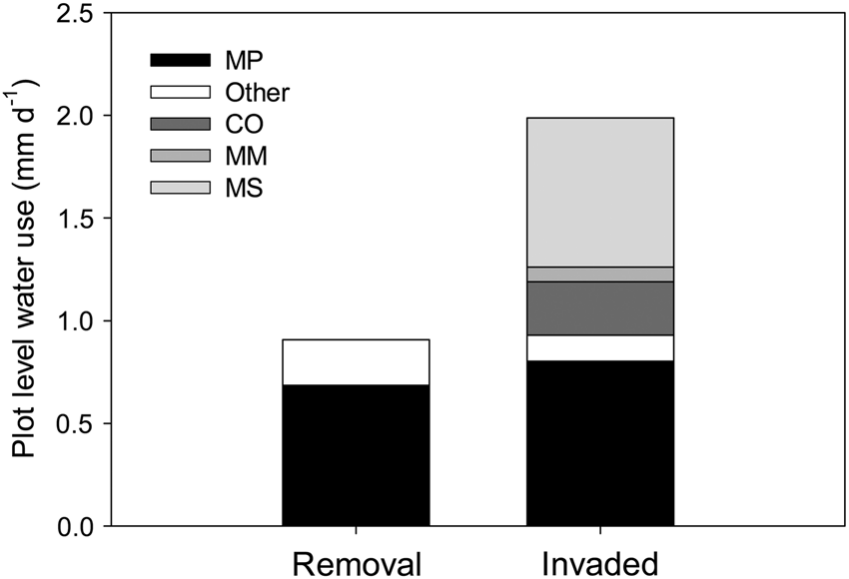
Estimated total plot-level transpiration by species for removal plots and invaded plots. See legend to Fig. 1 for species codes. Both native and non-native species not represented in the four instrumented species are referred to as ‘other’. Individual values for each species are as follows: removal plots, MP = 0.68 mm day^−1^ (248 mm year^−1^) and other natives = 0.22 mm day^−1^ (80 mm year^−1^); and invaded plots, MP = 0.80 mm day^−1^ (293 mm year^−1^), other natives = 0.04 mm day^−1^ (16 mm year^−1^), CO = 0.26 mm day^−1^ (29 395 mm year^−1^), MM = 0.07 mm day^−1^ (26 mm year^−1^), MS = 0.73 mm day^−1^ (265 mm year^−1^) and other invasives = 0.08 mm day^−1^ (30 mm year^−1^).

In the invaded forest plots, *Metrosideros* accounted for 40% of total water use because of the large size of the trees, while invasive *Melastoma* accounted for 37% of total water use because of the large number of stems per area (Fig. 7). Despite the very high sap-flow rates of *Macaranga* per unit sapwood area (Fig. 5B), the total contribution of this species was only 4% in the invaded plot because of low stem numbers (Fig. 1A). Plot-level transpiration decreased by 54% when all of the invasive species were removed, but total plot-level water use of the remaining *Metrosideros* was not greatly affected by removal of invasive species (Fig. 7). Compared with a mean annual precipitation rate of 3300 mm year^−1^ at this site prior to removal, the native *Metrosideros* were using ∼309 mm year^−1^ (9%), while the invasives were using ∼417 mm year^−1^ (13%) of total incoming rain (see Fig. 7 for individual species values).

## Discussion

### Native vs. invasive tree water use

Studies which directly compare co-occurring native vs. invasive tree transpiration rates in tropical forests are few, and results are mixed. Assumptions that invasive species use more water than their native counterparts are often based upon the belief that invasives grow faster than natives, and that fast-growing species often use more water. While one meta-analysis found invasiveness to be correlated with growth rate (van Kleunen *et al.*, 2010), others have found that invasive plants are no more likely to grow faster than co-ocurring natives, and that success depends largely on growing conditions (Daehler, 2003; Pyšek and Richardson, 2007). These broad results from meta-analyses suggest that commonly held assumptions about water use of invasive species are not always correct. For example, recent studies of assumed invasive ‘water spender’ saltcedar (*Tamarix ramosissima*) have shown that saltcedar transpires at similar rates compared with native cottonwoods and willows (Sala *et al.*, 1996; Devitt *et al.*, 1998; Cleverly *et al.*, 2002; Nagler *et al.*, 2003), but that its tolerance of drought, salt and fire enable it to out-compete natives very successfully (Glenn and Nagler, 2005). Likewise, fears that invasive melaleuca (*Melaleuca quinquenervia*) would cause widespread water draw-down throughout Florida's wetlands were found to be exaggerated, because previous assumptions were based upon erroneous extrapolation of pot studies to field conditions (Allen *et al.*, 1997). Additional studies in Australia have shown results contrary to expectation, where invasive willow species (*Salix* spp.) and native Red River Gum trees (*Eucalyptus camaldulensis*) had similar transpiration rates when growing on river banks (Doody and Benyon, 2011; Doody *et al.*, 2011). We set out to determine whether assumptions about native Hawaiian tree species' conservative water consumption compared with invasive trees would be supported by field evidence.

Determining the ecohydrological impact of invasive trees and the difference in water use between native and invading species is especially important throughout Hawaii, because most of the native wet forests on this archipelago are dominated by a single endemic species, *M. polymorpha.* These mono-dominant forests are also threatened throughout the islands by invasive species, the hydrologic consequences of which are unknown. In Hawaii, conventional wisdom that invasive tree species are ‘water spenders’ in comparison to co-occurring natives is based on little to no actual field measurements of transpiration at the tree scale or stand scale. While Hawaiian invasives had higher leaf-level stomatal conductance than natives in a Hawiian dry tropical forests (Cordell *et al.*, 2002), and a global meta-analysis found that invasives were more likely to have higher rates of stomatal conductance than co-occurring natives (Cavaleri and Sack, 2010), patterns of water use found at the leaf level are not necessarily applicable to whole-plant or ecosystem-level water use (Cavaleri and Sack, 2010). Without additional information about microclimate, canopy structure, sapwood area, leaf area index, interception and root architecture, it is very difficult to extrapolate leaf-level information to whole-tree transpiration and ecosystem evapotranspiration rates. We measured whole-tree transpiration directly via sap flux and were therefore able to extrapolate to the plot level to make meaningful large-scale comparisons.

As expected by our hypothesis, we have shown experimentally that *Metrosideros* was indeed more conservative in water use than all three measured invasive species, with lower peak and total daily sap-flow rates per unit sapwood area. Both vessel diameter and sapwood:basal area ratio of *Metrosideros* can vary greatly with elevational and rainfall gradients due to broad plasticity of the species (Fisher *et al.*, 2007; Santiago *et al.*, 2011). However, in comparison to other species on the islands, *Metrosideros* is one of the slowest growing, with the densest wood (corresponding to greater hydraulic resistance; Youngs, 1960), and both these factors are likely to have contributed to the low rates of transpiration per unit sapwood area found here. Our rates of whole-plant daily transpiration (2–25 kg day^−1^ in invaded plots and 5–43 kg day^−1^ in removal plots) were within the range previously reported for *Metrosideros* on Hawaii Island and Maui of 0.4–35 and 3–44 kg day ^−1^, respectively (Santiago *et al.*, 2000; Kagawa *et al.*, 2009). Kagawa *et al.* (2009) also found *Metrosideros* to use substantially less water per unit sapwood area than non-native species in nearby plantations of *Eucalyptus saligna* and *Fraxinus uhdei* in Hawaii. Kagawa *et al.* (2009), however, found whole-tree rates to be higher for the non-native species, which we did not find in the present study because our native tree diameters were much larger than invasive tree diameters (Fig. 1). Transpiration rates per unit sapwood of invasive species measured here (705–925 kg m^−2^_sw_ day^−1^; Fig. 5B) were on the high side compared with previously reported rates of tropical woody species. For *Eucalyptus globulus* and *Cupressus lusitanica* in a tropical montain forest in Ethiopia, mean daily rates per sapwood area were 200–400 kg m^−2^_sw_ day^−1^ (Fetene and Beck, 2004), compared with ∼500 kg m^−2^_sw_ day^−1^ for non-native plantations of *Fraxinus uhdei* and *Eucalyptus saligna* in Hawaii (Kagawa *et al.*, 2009). Per tree, however, our rates for daily water use of invasive species (4–6 kg day^−1^) were at least an order of magnitude lower than rates reported for other woody tropical species (e.g. Meinzer *et al.*, 2004; Andrade *et al.*, 2005; Kagawa *et al.*, 2009), probably due to the small diameters of the invasives in our plots. However, these small-diameter invasives will grow larger if left unchecked, and the forest of the future will probably show invasive whole-tree transpiration to be greater than native rates (e.g. Fig. 6).

### Effects of removal of invasive species on remnant natives

*Metrosideros* showed 16% higher sap-flow rates per unit sapwood area in removal plots than in the invaded plots. This effect was not consistent with our expectation of conservative resource use for this native species. This increase in water use was not associated with higher growth rates, however, as Ostertag *et al.* (2009) found no increase in remnant mature *Metrosideros* diameter growth 3 years after removal of the woody invasives in our study site. While it is not known how old the trees were in this study (growth rings do not correspond with annual growth in this aseasonal forest), *Metrosideros* is a very slow-growing species. Based on diameter growth rates of 1–2 mm year^−1^ (Ostertag *et al.*, 2009) and a DBH range of 90–420 mm, our instrumented *Metrosideros* probably span 45 to >400 years old. Others have found that the response of older trees to removal of invasives can be weaker than that of younger, more vigorously growing trees (Moore and Owens, 2012), and it is possible that 3 years post-removal is not an adequate time frame to evaluate impacts. Invasive grass removal has been shown slightly to increase *Metrosideros* growth in a dry tropical forest in Hawaii (D'Antonio *et al.*, 1998), but evidence shows that this was likely to be a release from nutrient competition rather than water competition, because soil moisture decreased after grass removal in this study, whereas available nitrogen increased.

Despite its conservative water use, *Metrosideros* can be sensitive to changes in water availability that occur during primary succession after lava flow and during advancement of invasion. For example, *Metrosideros* tends to be replaced by other native species, with succession from early pioneer communities on young lava flow systems to later seral communities, especially in dry forests of Hawaii, where *Metrosideros* is uncommon except on young lava flows (Stemmermann and Ihsle, 1993). Likewise, older sites (>300 years since lava flow) are generally more heavily invaded, with fewer surviving *Metrosideros* (Zimmerman *et al.*, 2008). Speculation in both cases is that *Metrosideros* is restricted due to limited water availability and increased competition. This evidence of sensitivity to water shortage, along with a history of periodic drought in these systems, suggests that invasives are more likely to impact remnant natives negatively through competition for water rather than competition for light or nutrients. Thus, increasing rates of periodic drought that may occur with climate change may exacerbate the adverse effects of invasive species on *Metrosideros*.

While *Metrosideros* did show a slight increase in water use after being released from competition, this increase did not compensate for the overall decrease in transpiration from the removed invasive species, leading to overall gain in water yield and thus potential aquifer recharge after removal. In a study of remnant oak savannas of the Midwestern USA, oaks increased transpiration rates by 42% following the removal of encroaching elms (Asbjornsen *et al.*, 2007), indicating both strong water limitation prior to removal and high physiological plasticity in the ability to respond to the increased water supply after removal. The enhancement of native water uptake after removal of invasive species may compensate for the decreased uptake by invasives, resulting in no net change in water use. This was the case in a removal study in a semi-arid riparian system in the Western USA, where removal of invasive salt cedar caused native cottonwood to increase transpiration rates to compensate, resulting in little change in overall water use between invaded and restored plots (Moore and Owens, 2012). The authors hypothesized that the invasive salt cedar had contributed relatively little to overall water flux prior to removal, due to low overall sapwood area and little available light in the shaded understory (Moore and Owens, 2012), but removal showed cottonwood to have been water limited in the invaded site. The capacity for increased transpiration after removal depends on the species, the initial water limitation of the native and the initial contribution of invasives to overall water flux.

Invasive species may acquire water from more shallow, more deep or the same soil depth as co-occurring natives; each case would result in differing responses in rooting depth or above- vs. below-ground allocation of native species. Removal of invasives may, in turn, cause natives to re-occupy the previously unavailable soil layers. In an Ethiopian tropical montane forest, non-native *Eucalyptus* were found to use water from deeper sources than the native co-occurring trees (Fritzsche *et al.*, 2006), suggesting that even if invasive trees do not use more water per unit sapwood, they may tap different water sources from natives, leading to very different patterns in groundwater use and overall system ecohydrology. A study of invasive grass removal in a dry tropical forest in Hawaii showed native trees to shift to more shallow water acquisition after removal of grasses, indicating direct competition of native woody species and invasive grasses in the shallow soil layers (Cordell and Sandquist, 2008). In our study, the natives and invasives are all woody and therefore more likely to be competing for the same root-growing space. Native vs. invasive competition for water could be exacerbated further in this site because soils are composed of young and unweathered lava substrate; therefore, water acquisition is likely to be confined to the uppermost soil layer (<0.5 m).

### Changes in plot-level water use and implications for restoration

Plot-level transpiration rates of the invaded vs. the removal plots were dramatically different in this lowland wet forest. The removal plots showed a 54% decrease in total plot-level tree transpiration or a difference of about 400 mm year^−1^, ∼12% of total incoming precipitation. While the fact that we were unable to collect sap-flux data before and after invasive species removal may introduce some error in our assessment of the effects of removal, we are confident that results would have been significant either way, given the magnitude of the response. This difference in plot-level water use between treatments should be interpreted as the potential gain in water yield after an invasive species removal programme, rather than the difference between native- vs. invasive-dominated systems. We do not know what the native subcanopy species were prior to invasion, and it is entirely possible that the invasives transpire as much water as the historical natives they replaced. The decreases in plot-level transpiration in the removal plots, therefore, were likely the result of an unoccupied niche from the large reduction in stem density (Doody *et al.*, 2011). The potential here for increase in aquifer recharge may justify the removal of invasives, if determined to be economically feasible. However, it is yet unknown what the long-term effects will be with respect to overall water use. Cordell *et al.* (2009) determined that this site has a very persistent invasive seed bank and could eventually revert back to being dominated by invasives without long-term follow-up management, including continued weeding and native inplanting.

The estimation of tree-level transpiration using the thermal dissipation method has well-documented errors associated with estimating the difference in voltage at minimal sap flux (Δ*V*_m_ in Equation 2) and also with using Granier's original empirical parameters (Steppe *et al.*, 2010). Estimation of individual tree sapwood area using allometric equations of DBH:sapwood thickness could also result in substantial error when scaling to the plot level, because the *R*^*2*^ values of these equations were quite low, especially for *Metrosideros* and *Melastoma* (Table 1). Additionally, we were unable to estimate or account for nocturnal water uptake (Oishi *et al.*, 2008) owing to the methodological challenges of irregular power supply, which could account for additional error. Despite these sources of error, the relative differences in transpiration rates between native and invasive trees are still relevant and significant, and the estimated differences in plot-level water use are likely to be conservative because, taken together, these artifacts of methodology tend to yield underestimations of sap-flux density (Steppe *et al.*, 2010).

Additional ecohydrological processes may also be impacted by this large removal of woody invasive basal area, including alterations in canopy interception, soil evaporation and the available energy for evaporation from leaves. Preliminary measurements of canopy interception were problematic owing to continued inundation at this extremely wet site and a relatively coarse weekly sampling schedule rather than a record of individual events (data not shown). Although we cannot speculate on the magnitude, the large removal of invasive leaf area probably did decrease total canopy interception, which probably increased the water yield and potential aquifer recharge in the removal plots, as the remaining native trees no longer provided a continuous canopy cover. Based on evapotranspiration maps of Hawaii, approximately one-quarter of total evapotranspiration is accounted for by canopy interception (Giambelluca *et al.*, 2014), and Safeeq and Fares (2014) found Hawaiian forests dominated by invasive tree species to intercept between 23 and 45% of incoming precipitation. Takahashi *et al.* (2011) found that the invasion of a *Metrosideros* forest resulted in more rainfall interception and less water available for aquifer recharge in comparison to the uninvaded forest. With respect to effects on soil evaporation, removal of invasive grasses in Hawaiian dry forests has been found both to increase (Thaxton *et al.*, 2012) and decrease soil moisture (D'Antonio *et al.*, 1998), indicating that results are dependent on site-specific attributes and methods of removal. In our study, soil evaporation was probably greater in the removal plot due to increased amplitude of both air and soil temperature as a result of increased exposure of the soil surface (Ostertag *et al.*, 2009); however, soil water potential was still found to be greater in the removal plots during times of drought (J. D. Michaud, unpublished observations). Soil evaporation for this site was estimated to be only ∼17% of total evapotranspiration, while transpiration was estimated to be ∼60% of evapotranspiration (Giambelluca *et al.*, 2014). Thus, even with a substantial decrease in plot-level transpiration after removal (Fig. 7), soil water potential measurements indicate that the decreased transpiration from removal plots may have compensated for any increased soil evaporation during dry periods. The decrease in total leaf area in the removal plots would also have decreased available energy, which probably contributed to the reduction in plot-level transpiration, because available energy is often the limiting factor to overall evapotranspiration in ecosystems where water is not limiting (Moore and Heilman, 2011).

To determine whether an invasive species removal programme will have the desired effect within a reasonable time frame calls for an ecosystem perspective and possibly a different valuation of resources, where potential water recharge is incorporated into the cost–benefit analysis of management options (Wise *et al.*, 2011). Unintended consequences and potential artifacts of removal include the release of additional undesirable species, removal of habitat for native fauna, alteration of trophic cascades, soil disturbance and compaction during treatment installation and monitoring (Aarssen and Epp, 1990; Campbell *et al.*, 1991; Zavaleta *et al.*, 2001). Despite these potential challenges to interpretation, removal experiments offer insight into complex species interactions and also help us to predict overall abiotic environmental responses to a management/restoration treatment that would mimic the experimental treatment. As in Hawaii, the supply of clean freshwater is decreasing throughout the world, while demand is simultaneously increasing with rising populations. In order to achieve long-term goals of water conservation in these and other systems, it is imperative that we understand the hydrological consequences of invasive species and the potential for restoration of invaded landscapes.

## Author contributions

M.A.C. and L.S. designed the sap-flow experiment. M.A.C. implemented the sap-flow experiment, analysed the data and wrote the manuscript. R.O. and S.C. designed and implemented the invasive removal experiment. R.O., S.C. and L.S. commented on the data analysis and manuscript at all stages.
